# Efficacy of leukocyte- and platelet-rich fibrin in the treatment and prevention of medication-related osteonecrosis of the jaw: a prospective study

**DOI:** 10.4317/medoral.27249

**Published:** 2025-05-27

**Authors:** Alejandro Medeiros-Monzón, Andrés Blanco-Carrión, Pilar Gándara-Vila, Gisela CV Camolesi, Alba Pérez-Jardón, Alejandro I Lorenzo-Pouso, Mario Pérez-Sayáns

**Affiliations:** 1 Oral Medicine, Oral Surgery and Implantology Unit (MedOralRes), Faculty of Medicine and Dentistry, University of Santiago de Compostela, Santiago de Compostela, Spain; 2 ORALRES Group, Health Research Institute of Santiago de Compostela (FIDIS), Santiago de Compostela, Spain; 3 Institute of Materials (IMATUS), Santiago de Compostela, Spain

## Abstract

**Background:**

Medication-related osteonecrosis of the jaws (MRONJ) is a serious condition associated with bone modifying agents (BMAs) intake, leading to impaired bone healing and increased morbidity. Despite various therapeutic approaches, an optimal treatment strategy remains elusive. Leukocyte- and Platelet- Rich fibrin (L-PRF) has emerged as a promising autologous biomaterial due to its regenerative properties. This study aimed to evaluate the efficacy of L-PRF in the treatment and prevention of MRONJ.

**Material and Methods:**

A prospective cohort study was conducted, including a total of 30 patients diagnosed with MRONJ (stage I or II) or at risk of developing it (non-MRONJ). Patient underwent standardized treatment involving surgical debridement followed by L-PRF application. Clinical and demographic data were collected, and healing outcomes were assessed at multiple follow-up intervals (7 days, 14 days, 1 month, 3 months and 6 months). Statistical analyses, including Kaplan-Meier survival estimates, were performed to evaluate treatment effectiveness.

**Results:**

The study demonstrated an overall healing of 90%, with a complete recovery in 82.4% of confirmed MRONJ cases and 100% of at-risk patients. L-PRF exhibited good clinical outcomes, including reduced inflammation and pain, accelerated epithelialization, and improved tissue regeneration. The median healing time was estimated at 33.41 days for MRONJ patients and 11.00 for non-MRONJ. No significant differences in healing rates were observed based on age, sex, or systemic conditions.

**Conclusions:**

L-PRF represents a promising adjunct in MRONJ management, improving healing outcomes and postoperative recovery. Its autologous nature and growth factor release enhance bone regeneration, suggesting its potential as both a therapeutic and preventive strategy. Further larger-scale clinical trials are needed to standardize protocols and validate long-term efficacy.

** Key words:**Medication-related osteonecrosis of the jaws, leukocyte- and platelet-rich fibrin, prevention, adjuvant therapies, bone healing.

## Introduction

**Figure 1:**
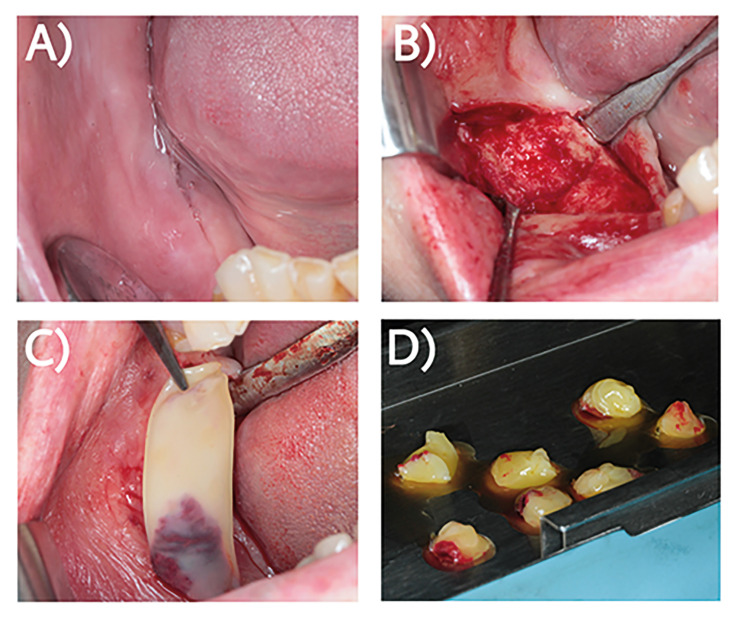
Surgical approach and application of L-PRF in a patient with MRONJ. (A) Initial clinical presentation of the affected area. (B) Surgical debridement of necrotic bone. (C) Application of L-PRF membrane over the surgical site. (D) Prepared L-PRF clots.

Osteonecrosis, also known as avascular necrosis, refers to the death of bone tissue due to insufficient blood circulation, which can lead to bone failure and joint disorders, ultimately becoming a significant source of disability in patients (1). This disease is characterized by the exposure of necrotic bone in the maxillofacial region that fails to heal within 8 weeks in patients treated with antiresorptive or antiangiogenic therapies, and who have not received radiation therapy to the craniofacial region (2) (Fig. 1). Several factors contribute to this condition, including surgery, trauma, corticosteroid intake, and alcoholism (3). Due to the increasing number of diagnosed cases, medication-related osteonecrosis of the jaws (MRONJ) has emerged as a major clinical concern (4).

Bisphosphonates, commonly used in the treatment of osteoporosis and metastatic bone disease, play a different role in the development of MRONJ by binding to hydroxyapatite in bone, inhibiting osteoclast-mediated bone resorption through induction of osteoclast apoptosis (5). Additionally, antiangiogenic agents further exacerbate this condition by inhibiting vascular endothelial growth factor, reducing angiogenesis and blood supply (6). Moreover, denosumab (i.e., a monoclonal antibody BMA), may also be part of these causative factors. While effective for managing conditions like osteoporosis and cancer, these mechanisms hinder bone repair and regeneration (7). As a result, MRONJ presents a major clinical problem, as it complicates the underlying disease and disrupts bone healing, negatively affecting the patient’s quality of life.

Given the substantial clinical challenges posed by MRONJ, the current lack of consistently effective management strategies highlights an urgent need for further research (8). Thus, understanding these differences is crucial for designing patient-specific treatment strategies and optimizing outcomes in both conditions.

To determine the most suiTable management options for MRONJ, it is essential to evaluate the efficacy and safety of various conservative treatments, surgical interventions, and biomaterials (9). For early-stage MRONJ, antibiotics and chlorhexidine rinses are commonly used, while surgical interventions—such as resection of necrotic bone (Fig. 1), microvascularized flap reconstruction, and neurolysis of the inferior alveolar nerve—are considered when conservative treatment fails (2,10).

Additionally, supportive therapies like alpha-tocopherol, pentoxifylline, ozone therapy, hyperbaric oxygen, and laser treatments (Erbium or low-level laser) have shown potential. Systemic administration of teriparatide, with or without local delivery of recombinant human bone morphogenic protein 2, has also shown promising results (11).

Among these strategies, Leukocyte- and Platelet-Rich Fibrin (L-PRF) stands out as a promising therapeutic option (12). As an autologous biomaterial derived from the patient’s own blood, it is minimally invasive and highly biocompatible. Unlike other platelet-rich products, L-PRF is particularly noted for its slow and sustained release of growth factors, which supports angiogenesis and osteogenesis (10).

L-PRF is obtained through the centrifugation of the patient’s whole blood without the addition of any additives or anticoagulants. Once centrifuged, it is necessary to separate the clot from the supernatant and the red blood cells. Then, it can be compressed into membranes (Fig. 1), making it versatile for clinical application (13). However, the technique used for preparing L-PRF can significantly impact treatment outcomes, and researchers have made significant progress in optimizing and standardizing these protocols to enhance L-PRF’s consistency and efficacy in clinical settings (10,14).

To address this complexity, L-PRF production requires linking its properties (clot morphology, size and structure) to factors like tube materials and blood phases. Centrifugation settings (g-force, time and acceleration) and storage methods (vacuum, homogenization and dehydration) must also be defined to ensure consistent clinical quality. Thus, standardizing these processes is essential to optimize L-PRF production and ensure consistent quality for clinical use (15).

Although preliminary studies have shown the potential of L-PRF to accelerate bone healing and reduce the increase of MRONJ (16), comprehensive clinical trials with larger sample sizes and standardized protocols are needed to validate its efficacy. Additionally, due to MRONJ variability (stage, affected site and patient comorbidities), treatment should be tailored, and evaluating L-PRF in conjunction with other therapies (antimicrobial treatment or photobiomodulation) is key to developing evidence-based guidelines.

Given this background, the primary objective of this prospective study is to evaluate the contribution of L-PRF in preventing and treating MRONJ.

## Material and Methods

- Study Design

This study was conducted as a prospective cohort aimed at evaluating the effectiveness of L-PRF in the prevention and treatment of MRONJ. The study protocol was approved by the Clinical Research Ethics Committee (code 301118), adhering to the principles outlined in the Declaration of Helsinki and its amendments and following the STROBE recommendations (17). All participants were fully informed about the procedures and provided written consent prior to their inclusion in the study. For the protection of confidentiality of the patient’s personnel details, a unique accession number was given on data extraction.

- Patient Selection and Clinical Data

This study included 30 participants who were prospectively recruited in the Oral Medicine, Oral Surgery and Implantology Unit of the University of Santiago de Compostela (USC). The patients included in this study were divided into two groups: those with a clinical diagnosis of stage I and II MRONJ (L-PRF was used as an adjuvant) and those at risk of developing it (L-PRF was used as a preventive treatment). The inclusion criteria comprised individuals of any gender undergoing treatment with BMAs. Patients were followed from the beginning of the study until the final data collection phase (with a minimum of 3 months). Exclusion criteria included individuals with osteonecrosis of a different origin, those with a history of radiotherapy and chemotherapy and patients with immunological disorders.

Each participant underwent a comprehensive clinical evaluation, which included a detailed medical history, a thorough oral examination, imaging studies, and, when deemed appropriate in the treatment, a surgical procedure through sequestrectomy of the necrotic bone followed by the application of L-PRF. The classification of MRONJ stages was based on the latest recommendations of the American Association of Oral and Maxillofacial Surgeons (AAOMS) (2).

Treatment decisions followed a standardized protocol established at the Oral Medicine, Oral Surgery, and Implantology Unit of the USC, based on the integrative review of L-PRF protocols by Salgado-Peralvo *et al*. (15). All treatment planning and surgical interventions were performed by the same highly experienced oral surgeon (M.P.S.).

Data were collected regarding the patient’s medical history, including level of oral hygiene, tobacco use, systemic conditions (cancer, osteoporosis), medication, type of antiresorptive drug, presence of coadjutants (e.g. corticosteroids), treatment duration, and surgical history. Next, the patients were evaluated over a follow-up period averaging 5.52 ± 1.35 months, during which information was gathered on colour, presence of inflammation, consistency, presence of granulation tissue, level of epithelialization, presence of purulence, presence of bleeding, and presence of pain in the treated area.

- Collection of L-PRF and Sample Processing

The collection and processing of L-PRF followed a standardized protocol (15) to ensure optimal preservation of its biological properties. Blood samples were obtained from each patient via venipuncture using BD Vacutainer® 21G x 3/4’’ x 7’’ (0.8mm x 19mm x 178mm) needles (Ref. 36782) and collected in INTRA-SPIN® tubes (Ref. BVBCTP, Sanhigia, Zaragoza, Spain) which are sterile, non-coated glasses tubes without anticoagulants to facilitate natural coagulation.

To prevent early clotting, all blood collections were performed in a controlled, sterile environment, and samples were transported promptly. After collection, blood samples were subjected to centrifugation at 2700 rpm for 12 minutes using a Uirimed® CNT800D angular analog centrifuge (Quirimed, Madrid, Spain) at room temperature. This process resulted in the formation of three layers: an upper acellular plasma layer, a middle L-PRF clot rich in platelets and leukocytes, and a lower layer composed of red blood cells. After centrifugation, the L-PRF clot was carefully extracted from the middle layer using sterile forceps, minimizing contamination from the red blood cell fraction. Subsequently, the clot was placed on a sterile metal grid or compression device (Fig. 1), where it was gently compressed using a sterile metal plate for approximately 5 minutes to form a membrane. This step aimed at expelling excess fluids while maintaining the structural integrity and biological activity of the fibrin network. Following membrane formation, the L-PRF samples were either used immediately in surgical applications or temporarily stored under sterile conditions. Short-term storage was performed in a controlled, humidified environment to prevent dehydration (15).

- Statistical Analysis

The statistical analysis was performed using SPSS version 26.0.1 (IBM Corporation, Armonk, NY, US). A univariate analysis was conducted, including the calculation of means, standard deviations, medians and interquartile ranges for continuous variables. For bivariate analysis, relationships between variables were examined using the Chi-square test for categorical variables, Fisher’s exact test when expected frequencies were below five, and the t-test for continuous variables with normal distribution. The Mann-Whitney U test was used for non-normally distributed continuous variables. To assess healing progression over time, a univariate survival analysis with the Kaplan-Meier estimator was conducted, and differences between groups were compared using the log-rank test. A statistician blinded to the data performed the hypothesis testing analysis. Significance level considered in all statistical analyses was 5% (*P* < 0.05).

## Results

- Sociodemographic and Clinical Characteristics of Participants

During the study duration, a total of 30 patients were identified who fulfilled the inclusion criteria. The sociodemographic and clinical characteristics of the study population are reported in Table 1. The mean age was 71.1 (SD= 9.2). Most participants had MRONJ (56.7%) with a higher proportion of women (63.3%) A smaller percentage were smokers (13.3%) and had poor oral hygiene (56.7%). The most prevalent conditions were cancer and osteoporosis. Most participants were poly-medicated (90%) and received adjuvant therapy (30% corticosteroids). The average duration of BMA treatment was 52.5 months, and the mean time without treatment before L-PRF therapy was 11.6 months. The size of MRONJ lesions varied widely, with a mean size of 90mm. Overall, 90% of participants experienced healing after treatment.

- Comparison of Sociodemographic and Clinical Characteristics Between Groups

Sociodemographic and clinical profiles of participants were compared dividing them into two groups (Table 2) stratified by the presence or absence of MRONJ. The age difference between MRONJ and non-MRONJ groups was minimal, with no significant statistical differences. Non-smokers were more common in MRONJ group (94.1%) than in non-MRONJ group (76.9%). Both groups had high poly-medication rates (non-MRONJ 76.9%, MRONJ 100%). Healing rates were slightly lower in the MRONJ group (82.4%) compared to non-MRONJ (100%).

- Healing Comparison Between Groups at Different Follow-Up Periods

Regarding the healing characteristics between the two groups at various follow-up intervals (7 days, 14 days, 1 month, 3 months, and 6 months) (Supplementary Table 1) initially, at 7 days, non-MRONJ participants had no redness, while 11.8% of MRONJ participants did. Both groups showed predominant pink coloration and minimal inflammation. Participants without MRONJ had firmer tissue, complete epithelialization, and better healing scores than those with MRONJ (61.5% excellent healing vs. 35.3%)

At 14 days, healing improved, with 92.3% of non-MRONJ achieving excellent healing versus 52.9% in the MRONJ group. Both groups showed continued pink coloration, minimal inflammation, and firmer tissue on palpation, but epithelialization was more advanced in the MRONJ group.

A similar trend was observed at the 1-month and 3-month follow-ups. At 6 months, non-MRONJ participants showed higher rates of complete epithelialization and excellent healing (100% vs. 84.6%) with both groups presenting firm, healed tissue and no signs of inflammation, suppuration or bleeding.

- Influence of Different Variables on Post - Surgical Healing

The different healing outcomes were compared across different variables (Table 3).

Healing rates were 100% for non-MRONJ and 82.4% for MRONJ, with no statistically significant differences between variables such as age, sex, tobacco use, oral hygiene, systemic diseases, or treatment duration. No significant correlations were found between surgical history, MRONJ size, or type of treatment and healing outcomes.

- Survival Analysis

**Figure 2:**
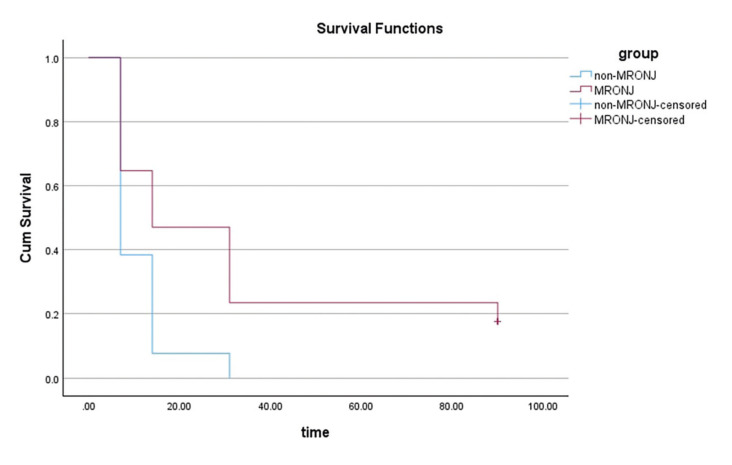
Survival analysis of MRONJ and non-MRONJ groups treated with L-PRF.

A survival analysis was conducted based on the healing time in patients with and without MRONJ (Fig. 2). Among the 30 cases included, 27 healing events were observed, with 3 cases censored. The mean healing time was longer in patients with MRONJ (33.41 days, 95% CI: 17.29-49.53) compared to those without MRONJ (11.00 days, 95% CI: 7.27-14.72). The Kaplan-Meier curve indicates a slower recovery in patients with MRONJ.

- Healing Score Comparison Between Groups

A comparative analysis of the healing scores between the groups, MRONJ and non-MRONJ, was conducted (Table 4). Healing was evaluated using the healing index (HI) by Hamzani *et al*. (18) where 1 indicates very poor healing and 5 represents excellent healing.

At 7-day follow-up, the non-MRONJ group exhibited a significantly higher mean healing score (4.5 ± 0.7) compared to the MRONJ group (3.7 ± 1.3) with a *p-value* = 0.052, suggesting a trend toward better healing in the non-MRONJ group. However, this difference did not reach statistical significance.

At 14-day follow-up both groups showed continued healing improvement, but the difference in mean healing scores remained (4.9 ± 0.3 in the non-MRONJ group vs. 4.3 ± 0.9 in the MRONJ group), with a *p-value* = 0.062, still indicating no statistical significance but a continued trend in favour of the non-MRONJ group.

At 1-month follow-up both groups achieved excellent healing, with the non-MRONJ group reaching an average score of 5.0 ± 0.0 and the MRONJ group scoring 4.8 ± 0.4. The difference between groups was not statistically significant (*p-value* = 0.235), suggesting that the gap in healing scores had reduced over time.

At 3 and 6 months, healing scores remained high in both groups, with no significant differences observed (mean scores of 5.0 ± 0.0 for non-MRONJ and 4.8 ± 0.4 for MRONJ at 3 months, and 5.0 ± 0.0 vs. 4.9 ± 0.4 at 6 months). The *p-value*s at these time points were 0.398 and 0.555, respectively, further indicating that both groups experienced similar healing outcomes as the follow-up period progressed.

## Discussion

In a prospective study involving 30 patients, the effectiveness of L-PRF in promoting healing of MRONJ-related lesions and preventing disease onset was evaluated. The findings demonstrated a healing rate of 90% over a six-month follow-up, with a 100% success rate in preventing MRONJ development. This finding highlights the therapeutic potential of L-PRF in improving the healing of tissues affected by MRONJ. The high success rate observed in this study reinforces the growing body of evidence supporting L-PRF as a viable approach particularly in cases where conventional treatments may have limited efficacy (19).

In the group of patients who did not have a clinical diagnosis of MRONJ but were considered at risk of developing this condition, treatment with L-PRF was favourable, achieving a 100% full recovery rate. Additionally, patients in this group showed an average healing score of 5 on the evaluation scale after 6 months. These results suggest that L-PRF may play a preventive role in modulating the progression of MRONJ in susceptible individuals, suggesting this technique not only as a therapeutic tool but also as a preventive measure in the management of this complication. This aligns with the hypothesis that L-PRF, through its autologous growth factor release and bioactive matrix, can enhance tissue resilience and promote faster repair, reducing the risk of osteonecrosis development (20,21).

Regarding the group of patients with a confirmed diagnosis of MRONJ, the observed healing rate was 82.4%, with an average healing score of 4.9 on the evaluation scale. Although the healing rate in these patients was slightly lower compared to the high-risk group without MRONJ, it remains a remarkable rate, especially considering the inherent difficulties in treating chronic and resistant bone lesions. These data reinforce the efficacy of L-PRF as a promising clinical intervention in complex scenarios of MRONJ, where conventional therapeutic options may not be as effective. The positive outcomes in MRONJ patients suggest that L-PRF may contribute to improved local vascularization and osteogenic activity, which are critical factors in reversing the compromised bone metabolism associated with MRONJ (22,23).

A significant aspect of this study was the observation of a notably faster postoperative recovery in patients treated with L-PRF. At 7 and 14 days after the procedure, there was substantial improvement in inflammation and pain parameters in patients receiving L-PRF. This finding suggests that the administration of L-PRF not only accelerates the healing process but also promotes a more comforTable postoperative recovery with a lower incidence of complications. The early reduction of inflammation and pain is a crucial factor in optimizing therapeutic outcomes and minimizing the impact of side effects on the patient’s quality of life. These benefits may be attributed to L-PRF’s fibrin network, which acts as a scaffold supporting the migration and proliferation of reparative cells while simultaneously reducing local inflammatory mediators (24).

The findings of this study are consistent with existing literature supporting the use of L-PRF in the management of MRONJ. Muñoz-Salgado *et al*. (25), in a systematic review and meta-analysis, found that L-PRF is highly effective in treating MRONJ and other conditions characterized by aberrant bone healing (26). Additionally, Pardo-Zamora *et al*. (10), in a cross-sectional study, demonstrated that L-PRF not only accelerated the healing process but also improved clinical outcomes for MRONJ patients, thereby supporting its use in this context. However, the variability in reported success rates across different studies suggests that patient selection criteria, L-PRF preparation protocols, and surgical techniques may influence treatment outcomes, highlighting the need to conform to standardized protocols (15).

Beyond MRONJ, the regeneration potential of L-PRF has been highlighted in various studies. Tenore *et al*. (27) reported an increase in the application of L-PRF for bone healing and regeneration. This is supported by the biological nature of the therapy, thereby confirming its potential for clinical use in stimulating tissue repair. Similarly, Ramos *et al*. (5) showed that L-PRF promotes wound healing by facilitating osteogenesis in oral and maxillofacial surgery.

In terms of its preventive potential, this study observed that 100% of at-risk patients who received L-PRF treatment achieved complete recovery. These findings parallel to previous reports indicating that L-PRF may play a protective role in preventing MRONJ development in susceptible individuals. Roman *et al*. (28) found that the application of L-PRF in patients undergoing invasive dental procedures while receiving bisphosphonates significantly reduced MRONJ incidence. Likewise, Inchingolo *et al*. (29) observed that the use of L-PRF helped preserve bone integrity and reduced the need for additional interventions.

On the other hand, the 82.4% healing rate observed in patients with a confirmed diagnosis of MRONJ in this study is slightly higher than that reported in previous research using similar approaches. Al-Hamed *et al*. (30) documented healing rates ranging from 75% to 80% in patients treated with L-PRF and surgical debridement, suggesting that factors such as surgical technique, graft preparation, and the frequency of platelet concentrate application may influence clinical outcomes. Further research should aim to elucidate the optimal parameters for L-PRF application, including the ideal concentration of growth factors, fibrin stability, and the synergistic effect with other regenerative therapies (31).

One of the main strengths of this study lies in its prospective design, which allows for detailed evaluation of the impact of L-PRF on the clinical progression of patients. Additionally, the standardization of the application protocol minimizes variability in the results, ensuring more consistent outcomes across the cohort. However, several limitations should be taken into account when interpreting the findings.

The relatively small sample size (30 patients) may have reduced the statistical power of the study, potentially limiting the ability to detect smaller differences or trends within the data. Additionally, the limited sample size restricts the generalizability of the results to larger, more diverse populations, which is an important factor when considering the widespread implementation of L-PRF in clinical practice.

Another limitation is the absence of a control group treated exclusively with conventional therapy. Without a control group, it is challenging to make a direct comparison between the effectiveness of L-PRF and other established therapeutic strategies.

Despite these limitations, the findings of this study suggest that L-PRF holds promise as a therapeutic and preventive tool for managing MRONJ, with its potential to accelerate tissue healing and improve postoperative recovery. Future studies with larger sample sizes, well-defined control groups, and longer follow-up periods will be essential to further assess the efficacy if L-PRF in diverse patient populations and its long-term benefits in preventing and treating MRONJ. These studies would help clarify the full scope of its clinical applications and validate its place in the therapeutic arsenal for MRONJ management.

In summary, this study suggests that L-PRF may serve as both a therapeutic and preventive strategy for MRONJ. The high healing rates and improved postoperative recovery indicate its potential to enhance tissue repair and modulate bone metabolism. Incorporating L-PRF into MRONJ treatment protocols could optimize outcomes, especially in at-risk patients, by promoting early tissue regeneration and preventing disease progression.

## Conclusions

This study underscores the potential of L-PRF in advancing MRONJ treatment, showing a high healing rate of 82.4% in confirmed MRONJ cases and 90% in the overall cohort. The 100% recovery in at-risk patients highlights its promise in both preventing and treating MRONJ progression. However, further research is needed to refine L-PRF protocols, such as optimal frequency, dosage, and its combination with other regenerative therapies. Larger-scale, multicenter clinical trials are essential to confirm L-PRF’s long-term efficacy in both treating and preventing MRONJ.

## Figures and Tables

**Table 1:** Sociodemographic and Clinical Characteristics of the Study Participants (n=30).

Characteristics		(Mean ± SD) / N	Median (IQR) / (%)
Age		71.1 ± 9.2	72 (64 - 76)
Group	Non-MRONJ	13	(43.3%)
	MRONJ	17	(56.7%)
Sex	Male	11	(36.7%)
	Female	19	(63.3%)
Tobacco	Do not smoke	26	(86.7%)
	Smoke	4	(13.3%)
Oral Hygiene	Poor	17	(56.7%)
	Moderate	8	(26.7%)
	Good	5	(16.7%)
Systemic diseases	Other diseases	1	(3.3%)
	Cancer	15	(50.0%)
	Osteoporosis	12	(40.0%)
Medication	Drug	3	(10.0%)
	Polimedication	27	(90.0%)
Antiresorptive drug	Intravenous	8	(26.7%)
	Oral	4	(13.3%)
	Subcutaneous	9	(30.0%)
	Combination BMAs	4	(13.3%)
Coadjuvant	No	21	(70.0%)
	Corticosteroids	9	(30.0%)
Duration of treatment		52.5 ± 69.4	24 (6 - 78)
Time since treatment		11.6 ± 14.7	5 (2 - 16)
Surgical History	No	13	(43.3%)
	Yes	17	(56.7%)
Puncture zone	Right	11	(36.7%)
	Left	10	(33.3%)
	Right + Left	2	(6.7%)
Tubes		5.2 ±2.3	4 (4 - 8)
Plugs		4.9 ± 2.8	4 (3 - 8)
Membranes		1.5 ± 1.2	1 (1 - 1)
Localization	1st quadrant	8	(26.7%)
	2º quadrant	2	(6.7%)
	3º quadrant	8	(26.7%)
	4º quadrant	5	(16.7%)
	Both quadrants simultaneously	7	(23.3%)
Fragment mobility	No	15	(50.0%)
	Yes	4	(13.3%)
	Non-MRONJ	8	(26.7%)
Size of MRONJ		174.3 ± 247.8	90 (300)
Fistula	No	21	(70.0%)
	Yes	6	(20.0%)
Treatment	Surgery + antibiotics	30	(100.0%)
Cure	Yes	27	(90.0%)
	No	3	(10.0%)

**Table 2:** Comparison of Sociodemographic and Clinical Characteristics of Participants Between the Two Groups.

Characteristics		Group				P value
		Non- MRONJ		MRONJ		
		(Mean ± SD) / N	Median (IQR) / (%)	(Mean ± SD) / N	Median (IQR) / (%)	
Age		69.8 ± 7.8	70 (64 - 73)	72 ± 10.3	74 (63 - 79)	0.271
Sex	Male	6	(46.2%)	5	(29.4%)	0.346
	Female	7	(53.8%)	12	(70.6%)	
Tabacco	Do not smoke	10	(76.9%)	16	(94.1%)	0.290
	Smoke	3	(23.1%)	1	(5.9%)	
Oral Hygiene	Poor	10	(76.9%)	7	(41.2%)	0.145
	Moderate	2	(15.4%)	6	(35.3%)	
	Good	1	(7.7%)	4	(23.5%)	
Systemic diseases	Other diseases	0	(0.0%)	1	(6.7%)	0.246
	Cancer	9	(69.2%)	6	(40.0%)	
	Osteoporosis	4	(30.8%)	8	(53.3%)	
Medication	Drug	3	(23.1%)	0	(0.0%)	0.070
	Polimedication	10	(76.9%)	17	(100.0%)	
Antiresorptive drug	Intravenous	4	(50.0%)	4	(23.5%)	0.133
	Oral	0	(0.0%)	4	(23.5%)	
	Subcutaneous	4	(50.0%)	5	(29.4%)	
	Combination BMAs	0	(0.0%)	4	(23.5%)	
Coadjuvant	No	10	(76.9%)	11	(64.7%)	0.469
	Corticosteroids	3	(23.1%)	6	(35.3%)	
Duration of treatment		24.7 ± 25.6	24 (3 - 24)	62.94 ± 78	24 (6 - 96)	0.266
Time since treatment		18.8 ± 20.8	14 (6 - 16)	9.1 ± 11.6	3 (2 - 12)	0.138
Surgical History	No	9	(69.2%)	4	(23.5%)	0.012
	Yes	4	(30.8%)	13	(76.5%)	
Puncture zone	Right	7	(70.0%)	4	(30.8%)	0.135
	Left	3	(30.0%)	7	(53.8%)	
	Right + Left	0	(0.0%)	2	(15.4%)	
Tubes		5.3 ± 2.7	5 (3 - 8)	5.2 ± 1.9	4 (4 - 8)	0.969
Plugs		5.7 ± 2.9	8 (3 - 8)	4.0 ± 2.7	4 (2 - 6)	0.240
Membranes		1.0 ± 0	1 (1 - 1)	2.0 ± 2.0	1 (1 - 3)	0.800
Localization	1st quadrant	3	(23.1%)	5	(29.4%)	0.403
	2º quadrant	0	(0.0%)	2	(11.8%)	
	3º quadrant	3	(23.1%)	5	(29.4%)	
	4º quadrant	2	(15.4%)	3	(17.6%)	
	Both quadrants simultaneously	5	(38.5%)	2	(11.8%)	
Fragment mobility	No	2	(20.0%)	13	(76.5%)	-
	Yes	0	(0.0%)	4	(23.5%)
	Non-MRONJ	8	(80.0%)	0	(0%)
Size of MRONJ		Non-MRONJ		276.9 ± 263.8	90 (300)	-
Fistula	No	10	(100.0%)	11	(64.7%)	0.057
	Yes	0	(0.0%)	6	(35.3%)	
Treatment	Surgery + antibiotics	13	(100.0%)	17	(100.0%)	1.000
Cure	Yes	13	(100.0%)	14	(82.4%)	0.238
	No	0	(0.0%)	3	(17.6%)	

**Table 3:** Comparison of Post Surgical - Healing among Different Variables.

Characteristics		Healed				P value
		YES	NO			
		(Mean ± SD) / N	Median (IQR) / (%)	(Mean ± SD) / N	Median (IQR) / (%)	
Group	Non-MRONJ	13	(100.0%)	0	(0.0%)	0.238
	MRONJ	14	(82.4%)	3	(17.6%)	
Sex	Male	10	(90.9%)	1	(9.1%)	1.000
	Female	17	(89.5%)	2	(10.5%)	
Age		71.2 ± 9.6	73 (64 - 78)	70.0 ± 6.2	72 (63 - 75)	-
Tabacco	Do not smoke	24	(92.3%)	2	(7.7%)	0.360
	Smoke	3	(75.0%)	1	(25.0%)	
Oral Hygiene	Poor	15	(88.2%)	2	(11.8%)	0.732
	Moderate	8	(100.0%)	0	(0.0%)	
	Good	4	(80.0%)	1	(20.0%)	
Systemic diseases	Other diseases	1	(100.0%)	0	(0.0%)	0.524
	Cancer	13	(86.7%)	2	(13.3%)	
	Osteoporosis	12	(100.0%)	0	(0.0%)	
Medication	Nothing	0	(0.0%)	0	(0.0%)	1.000
	Drug	3	(100.0%)	0	(0.0%)	
	Polimedication	24	(88.9%)	3	(11.1%)	
Antiresorptive drug	Intravenous	6	(75.0%)	2	(25.0%)	0.624
	Oral	4	(100.0%)	0	(0.0%)	
	Subcutaneous	8	(88.9%)	1	(11.1%)	
	Combination BMAs	4	(100.0%)	0	(0.0%)	
Coadjuvant	No	19	(90.5%)	2	(9.5%)	1.000
	Corticosteroids	8	(88.9%)	1	(11.1%)	
Duration of treatment		53.5 ± 71.8	24 (6 - 72)	46.0 ± 64.2	12 (6 - 120)	-
Time since treatment		11.5 ± 15.4	5 (3 - 16)	12.7 ± 11.0	12 (2 - 24)	-
Surgical History	No	12	(92.3%)	1	(7.7%)	1.000
	Yes	15	(88.2%)	2	(11.8%)	
Puncture zone	Right	10	(90.9%)	1	(9.1%)	1.000
	Left	9	(90.0%)	1	(10.0%)	
	Right + Left	2	(100.0%)	0	(0.0%)	
Tubes		5.3 ± 2.3	4 (4 - 8)	4.0 ± 0.0	4 (4 - 4)	-
Plugs		4.9 ± 2.8	4 (3 - 8)	-		-
Membranes		1.5 ± 1.2	1 (1 - 1)	-		-
Localization	1st quadrant	8	(100.0%)	0	(0.0%)	0.123
	2º quadrant	2	(100.0%)	0	(0.0%)	
	3º quadrant	8	(100.0%)	0	(0.0%)	
	4º quadrant	3	(60.0%)	2	(40.0%)	
	Both quadrants simultaneously	6	(85.7%)	1	(14.3%)	
Fragment mobility	No	12	(80.0%)	3	(20.0%)	0.549
	Yes	4	(100.0%)	0	(0.0%)	
	Non-MRONJ	8	(100.0%)	0	(0.0%)	
Size of MRONJ		269.4 ± 285.2	160 (90 - 345)	312.3 ± 160.6	297 (160 - 480)	-
Fistula	No	18	(85.7%)	3	(14.3%)	1.000
	Yes	6	(100.0%)	0	(0.0%)	
Treatment	Surgery + antibiotics	27	(90.0%)	3	(10.0%)	1.000

**Table 4:** Comparative Healing Scores Between Patients with and without MRONJ.

Total	Overall	Group				P value
		Non-MRONJ		MRONJ		
	Mean ± SD	Mean ± SD	Median (IQR)	Mean ± SD	Median (IQR)	
7d	4.0 ± 1.2	4.5 ± 0.7	5.0 (4.0 - 5.0)	3.7 ± 1.3	4.0 (3- 5.0)	0.052
14d	4.6 ± 0.7	4.9 ± 0.3	5.0 (5.0 - 5.0)	4.3 ± 0.9	5.0 (4.0 - 5.0)	0.062
1m	4.9 ± 0.3	5.0 ± 0.0	5.0 (5.0 - 5.0)	4.8 ± 0.4	5.0 (5 - 5.0)	0.235
3m	4.9 ± 0.3	5.0 ± 0.0	5.0 (5.0 - 5.0)	4.8 ± 0.4	5.0 (5.0 - 5.0)	0.398
6m	4.9 ± 0.3	5.0 ± 0.0	5.0 (5.0 - 5.0)	4.9 ± 0.4	5.0 (5.0 - 5.0)	0.555
